# SARS-CoV-2 Y453F is not the “cluster 5” variant

**DOI:** 10.1016/j.jbc.2021.101242

**Published:** 2021-10-23

**Authors:** Ria Lassaunière

**Affiliations:** Virus and Microbiological Special Diagnostics, Statens Serum Institut, Copenhagen, Denmark

I read with interest the recent publication by Bayarri-Olmos *et al.* (1) and would like to express some concerns regarding the study.

The SARS-CoV-2 receptor-binding domain (RBD) variant investigated by Bayarri-Olmos *et al.* (1), Y453F, is not the “cluster 5” variant (2). We initially described the “cluster 5” variant (3, 4), which has five spike amino acid changes (Fig. 1; strain: hCoV-19/Denmark/DCGC-3024/2020; GISAID number: EPI_ISL_616802). The mislabeling and out-of-context comparison of the Y453F properties with that of the multispike mutation variant “cluster 5” are incorrect and misleading. This kind of reporting is not only careless, but also irresponsible in light of the ongoing disputes surrounding the mink culling in Denmark. Furthermore, the “cluster 5” preliminary report clearly states that a microneutralization assay with an antinucleocapsid ELISA read-out was used (3), not a “*bona fide* plaque reduction neutralization assay.” This further casts into doubt the thoroughness by which the authors studied the cited report and their understanding of SARS-CoV-2 virus neutralization assays.

**Figure 1:**
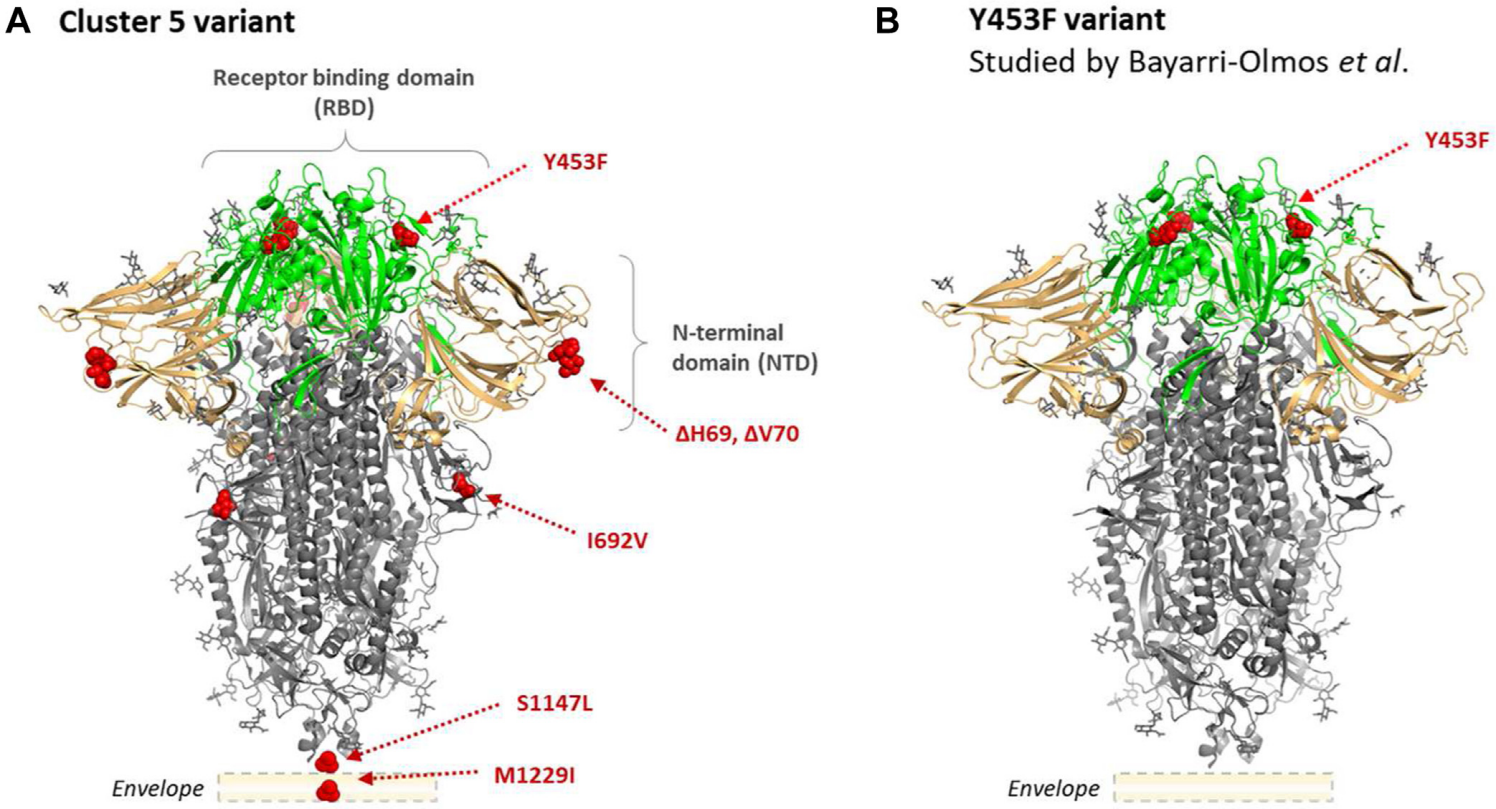
**The crystal structure of a closed prefusion SARS-CoV-2 spike trimer [PDB:****6ZGE****] indicating the amino acid changes oftwo different variants.***A*, the SARS-CoV-2 cluster 5 variant described in a preliminary report by Statens Serum Institut (3, 4); and *B*, the SARS-CoV-2 Y453F receptor-binding domain (RBD) variant studied by Bayarri-Olmos *et al.* (1). *Red spheres* indicate the positions of amino acid changes. The RBD is presented in *green*, the N-terminal domain (NTD) in *beige*, and the S2 domain in *gray*. The regions encompassing the S1147L and M1229I substitutions are not within the crystal structure; however, their relative positions are indicated.

The Y453F variant has some degree of neutralization resistance (5). Due to the heterogeneity of antibody responses, mutations will differentially affect convalescent plasma. It is therefore relevant to investigate the effect of the Y453F variant on an individual level and not only grouped. Lastly, the authors expressed surprise upon the observed higher affinity of the 453F RBD for ACE2 compared with the wild-type. However, this finding is not novel. Starr *et al.* (6) already demonstrated enhanced binding of the Y453F RBD to human ACE2 in September 2020.

## Conflict of interest

The author declares no conflicts of interests with the contents of this article.
